# Thermo-Mechanical Analysis for Composite Cylindrical Shells with Temperature-Dependent Material Properties Under Combined Thermal and Mechanical Loading

**DOI:** 10.3390/ma18071391

**Published:** 2025-03-21

**Authors:** Junjie Li, Hai Qian, Chunhua Lu

**Affiliations:** Faculty of Civil Engineering and Mechanics, Jiangsu University, Zhenjiang 212013, China; 2212223025@stmail.ujs.edu.cn (J.L.); lch79@ujs.edu.cn (C.L.)

**Keywords:** laminated cylindrical shell, Fourier’s law of heat conduction, temperature dependence, theory of thermoelasticity, state-space method

## Abstract

Composite laminated structures, comprising various engineering materials, are extensively utilized in engineering structures due to their superior design flexibility and enhanced mechanical performance. This study investigates the mechanical behavior of laminated cylindrical shells under combined thermal and mechanical loads. Using the theory of thermoelasticity, exact solutions are derived for temperature, displacement, and stress distributions in axisymmetric cylindrical shells with arbitrary numbers of layers and varying thicknesses, considering the temperature-dependent properties of the component materials. An iterative method and a slice model are introduced to address the interplay between temperature variations and material properties with the transfer matrix method on the basis of Fourier’s law of heat conduction. Stresses and displacements are used to formulate the state-space equation. Continuity conditions at the interfaces are applied to recursively establish the relationships between internal and external surfaces by the state-space method. Unique solutions for temperature, displacement, and stress, which are dependent on temperature, are determined by the surface conditions. The high accuracy and effectiveness of the proposed method are validated through convergence and comparative studies. Notably, neglecting temperature dependence leads to significant differences, with temperature increasing by 11.28%, displacement by 17.35%, and stress by 33.74%. Furthermore, the effects of surface temperature, thickness-to-radius ratio, layer numbers, and component materials on the temperature, displacement, and stress distributions within laminated cylindrical shells are thoroughly explored.

## 1. Introduction

Composite laminated cylindrical shells, composed of multiple engineering materials, have wide-ranging applications in the field of engineering structures due to their exceptional design flexibility and mechanical performance. These structures are widely applied in various domains, including building structures, transportation infrastructure, and bridge engineering, such as vacuum pipes for wheel–rail trains, pipelines for offshore oil and gas transportation, drainage pipes in municipal sewage systems, and the fuselage skin in the aerospace sector [1,2,3,4,5,6]. When subjected to thermal–mechanical loads, laminated cylindrical shells exhibit non-uniform displacements and stresses, potentially causing structural failure and damage, such as thermal cracking in oil pipelines and heat-induced damage to vacuum pipes during train operation. Analyzing the behaviors of laminated structures under simultaneous temperature and mechanical loads is essential for maintaining structural safety [7,8,9,10]. However, achieving accurate solutions for displacement and stress within laminated cylindrical shells, when subjected to thermo-mechanical loads, presents significant challenges due to the complex interdependencies between material properties and temperature variations.

Over the years, the mechanical behavior of laminated structures has been extensively studied [11,12]. The primary analytical theories are categorized as the equivalent single-layer theory [13], the zig-zag theory [14], and the layer-wise theory [15]. Embebbed within the equivalent single-layer theory, the method is subclassified into the classical laminate theory, the first-order shear deformation theory, and the higher-order shear deformation theory, each differentiated by the specific assumption made regarding the displacement changes throughout the laminate thickness. The classical laminate theory [16] is predicated upon Kirchhoff’s linear hypothesis, which postulates that the cross-sectional shape maintains a planar configuration and remains its orientation relative to the neutral axis throughout the entire deformation process. Mirzavand and Pourmohammad [17] explored the post-buckling behavior of functionally graded shells under thermal conditions according to the classical laminate theory. He et al. [18] developed a finite element model to control the vibration and shape of plates, based on the principles of classical laminate theory. Ghasemi et al. [19] introduced a new algorithm for calculating residual stresses within the framework of the classical laminate theory and studied how temperature variations and thermal cycling affect residual stresses in composite structures. The classical laminate theory delivers precise results for thin, isotropic structures. However, neglecting shear deformation across the thickness can result in inaccuracy in analyzing the laminated structures with substantial thickness or marked in-plane anisotropic properties. The first-order shear deformation theory improves upon the classical laminate theory by incorporating shear deformation across the thickness. In accordance with this theory, the cross-section remains planar during deformation, despite not aligning perpendicularly with the neutral axis. Based on the first-order shear deformation theory, Topal and Uzman [20] investigated the thermal buckling responses of symmetric laminated cylindrical shells under uniformly distributed temperature loads. Tornabene et al. [21] employed the first-order shear deformation theory to examine the structural components of functionally graded conical shells and annular plates with moderate thickness. Bouderba et al. [22] validated the thermal buckling behavior of sandwich plates with functionally graded materials under different boundary conditions using the first-order shear deformation theory. The first-order shear deformation theory incorporates transverse shear deformation by assuming that shear strain remains uniform throughout the laminate thickness. Incorporating a shear correction factor is crucial for ensuring the alignment of shear stress with the surface boundary conditions of the laminated structure. Additionally, the higher-order displacement distribution function has been employed to model in-plane displacements, improving the accuracy of mechanical analysis of laminated structures. Commonly employed displacement distribution functions encompass cubic polynomials, sine functions, exponential functions, logarithmic functions, hyperbolic functions, and their combinations [23,24]. Ramezani et al. [25] applied the higher-order shear deformation theory to conduct the nonlinear response of laminated cylindrical shells exposed to mechanical and thermal conditions. By applying the higher-order shear deformation theory, Safarpour et al. [26] employed an analytical study of free vibration in graphene flat plates. The higher-order shear deformation theory is extensively applied for the analysis of displacements and stresses within laminated structures. Nonetheless, this theory posits discontinuities in stresses at the interfaces, complicating the precise assessment of interlaminar and transverse shear stress.

The zig-zag theory [27,28,29] constructs zig-zag displacement functions for each layer, which satisfies the continuities of interlayer displacement and transverse shear stress in advance. Padhi and Pandit [30] investigated the behavior of laminated sandwiches subjected to thermal loading with the higher-order zig-zag theory. Ghalami-Choobar et al. [31] carried out an investigation of the static behavior of short hybrid laminated beams through the application of the zig-zag theory. Icardi [32] developed a sublaminar model for analyzing laminated beams using the zig-zag theory to evaluate the practical benefits of higher-order approximations to sublaminar internal displacements. The zig-zag theory, due to its higher computational accuracy relative to higher-order theory with fewer unknown displacement quantities, is therefore widely utilized for analyzing the static behavior of laminated structures. Nevertheless, the zig-zag theory does not consider the effect of transverse normal strain. Additionally, boundary conditions at the edges of laminated structures for shear stress in the transverse direction are not satisfied by this theory.

The layer-wise theory [33,34,35] divides the laminated structure into several sublayers along the thickness and assumes that the displacement field has separate expansion formulas within each sublayer. Tornabene et al. [36] developed a generalized approach using the layer-wise theory for performing linear static analyses of doubly curved shells. Fares et al. [37] introduced the layer-wise theory to examine the bending and vibrational responses of multilayer hyperbolic shells. The layer-wise theory is effective in representing the independent deformation of each layer within the structure. However, unknown variables are influenced by the quantity of sublayers. As the layer number increases, the variables of the function will correspondingly increase, significantly raising the computational cost.

The thermoelastic behavior of axisymmetric laminated cylindrical shells is studied in this paper, considering the material properties in relation to temperature and utilizing the theory of thermoelasticity. In light of the interplay between temperature within the shell and material properties, an iterative method and a slice model are introduced to ascertain the exact temperature distribution within the structure. Displacements and stresses are used to formulate the state-space equation. Leveraging the continuity conditions between neighboring layers, the relationships of the stresses and displacements between the external and internal surface layers are established by the transfer matrix method. Combined with the surface conditions, the unique solutions for displacement and stress in shells made from temperature-dependent materials are obtained. The presented methodology is computationally efficient, ultimately reduced to solving two linear equations, with its effort not influenced by the number of material layers in the structure. This approach is applicable for analyzing displacements and stresses in laminated shells of arbitrary component materials, thicknesses, and layer numbers within a temperature environment.

## 2. Geometrical Model

**Figure 1:**
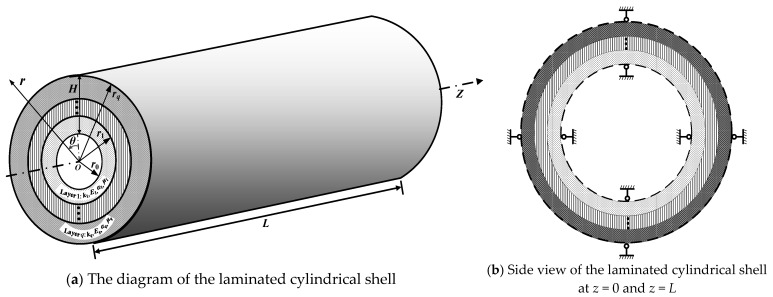
The schematic diagram of the laminated cylindrical shell.

A simply supported laminated cylindrical shell is illustrated in Figure 1. The total number of material layers is represented as *q*. The internal radius and the external radius are *r*_0_, and *r_q_*. The length of the shell is *L* and the thickness is defined as *H* (*H* = *r_q_* − *r*_0_). The outer radius of each single layer is, respectively, denoted as *r_i_* (*i* = 1, 2, …, *q*). The thickness of each material layer in the laminated cylindrical shell is *H_i_* (*H* = *r_i_ − r_i_*_−1_). The thermal conductivity *k_i_*, the Poisson’s ratio *μ_i_*, the thermal expansion coefficient *α_i_*, and the elastic modulus *E_i_* are the material properties of the *i*th layer. Temperature and mechanical loads are simultaneously considered on laminated cylindrical shells. The temperature at the internal and external surfaces of the shell is *t*_0_ and *t_q_*, as follows:

(1) $$T\left(r_{0}\right)=t_{0},\;T\left(r_{q}\right)=t_{q}.$$

The shell surfaces are subjected to the axisymmetrically distributed loads *Q_in_*(*z*) and *Q_ex_* (*z*), as follows:

(2) $$\sigma_{r}\left(r_{0},z\right)=Q_{in}\left(z\right),\;\sigma_{r}\left(r_{q},z\right)=Q_{ex}\left(z\right).$$

## 3. Analysis of the Temperature Field

Due to the combined temperature and mechanical loads, the material properties of the cylindrical shell are consistently influenced by environmental temperature variations. Given the intricate temperature distribution within the laminated shell, which comprises temperature-dependent materials, obtaining temperature solutions that are independent of material properties is initially practical.

### 3.1. Temperature Solutions for Temperature Independence

The equation for heat conduction along the *r*-direction of the cylindrical coordinate system can be expressed as [38]

(3) $$\left(\frac{d^{2}}{dr^{2}}+\frac{1}{r}\frac{d}{dr}\right)T_{i}\left(r\right)=0.$$

The temperature solution of Equation (3) can be obtained as

(4) $$T_{i}\left(r\right)=A_{i}+B_{i}\ln\left(r\right),$$

where *A_i_* and *B_i_* are the undetermined coefficients of the *i*th layer.

To simplify, the general temperature solution and the heat flux are presented in matrix form as follows:

(5) $$\left[\begin{array}{c} T_{i}\left(r\right)\\ k_{i}\frac{\partial T_{i}\left(r\right)}{\partial r} \end{array}\right]=\left[\phi_{i}\left(r\right)\right]=\left[\varphi_{i}\left(r\right)\right]\left[\Omega_{i}\right],$$

(6) $$\left[\varphi_{i}\left(r\right)\right]=\left[\begin{array}{cc} 1 & \ln\left(r\right)\\ 0 & \frac{k_{i}}{r} \end{array}\right],\;\left[\Omega_{i}\right]=\left[\begin{array}{c} A_{i}\\ B_{i} \end{array}\right].$$

Taking *r* = *r_i−_*_1_ and *r* = *r_i_* (*i* = 2, 3, …, *q*) into Equation (5), the temperature and heat flux of the inside and outside surface of *i*th layer can be given by

(7) $$\left[\phi_{i}\left(r_{i}\right)\right]=\left[\varphi_{i}\left(r_{i}\right)\right]{\left[\varphi_{i}\left(r_{i-1}\right)\right]}^{-1}\left[\phi_{i}\left(r_{i-1}\right)\right].$$

The temperature and heat flux at the interface of neighboring layers are continuous, i.e.,

(8) $$\left[\phi_{i}\left(r_{i}\right)\right]=\left[\phi_{i-1}\left(r_{i}\right)\right].$$

The relationships of the undetermined coefficients for the *p*th (*p* = 2, 3, …, *q*) layer and the first layer can be obtained by the following recursion from Equations (7) and (8), as follows:

(9) $$\left[\Omega_{p}\right]={\left[\varphi_{p}\left(r_{p}\right)\right]}^{-1}\left\{\prod_{j=p}^{1}\left[\varphi_{j}\left(r_{j}\right)\right]{\left[\varphi_{j}\left(r_{j-1}\right)\right]}^{-1}\right\}\left[\varphi_{1}\left(r_{0}\right)\right]\left[\Omega_{1}\right].$$

Substituting the internal and external surface temperature (1) into Equation (4) yields

(10) $$t_{0}=A_{0}+B_{0}\ln(r_{0}),\;t_{q}=A_{q}+B_{q}\ln\left(r_{q}\right).$$

The relationships of undetermined coefficients between the external and internal layers of the laminated shell are followed by substituting *p* = *q* into Equation (9). The undetermined coefficients *A*_0_, *B*_0_, *A_q_*, and *B_q_* of the internal and external layers can be obtained by solving Equations (9) and (10) in tandem. Taking the coefficients *A*_0_ and *B*_0_ into the recurrence relation (9), the undetermined coefficients *A_i_* and *B_i_* (*i* = 1, 2, …, *q*) for all layers within the shell can be ascertained. Finally, the analytical temperature solution for the laminated cylindrical shell with temperature-independent materials is uniquely determined.

### 3.2. Temperature Analysis for Temperature Dependence

A slice model and an iterative method are introduced to investigate the temperature distribution for the laminated shell with temperature-dependent materials in this section.

#### 3.2.1. The Slice Model

**Figure 2:**
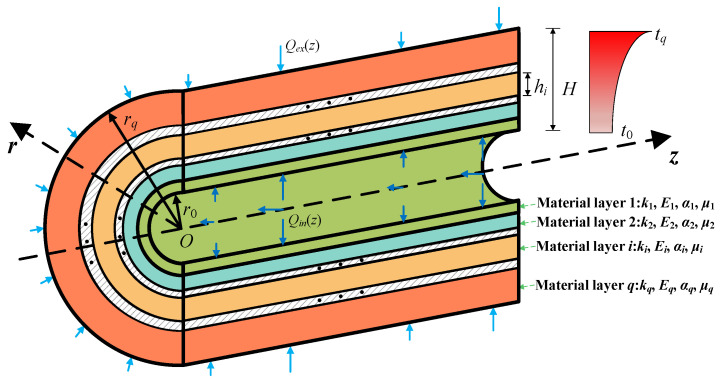
The cross-section view of the laminated cylindrical shell.

**Figure 3:**
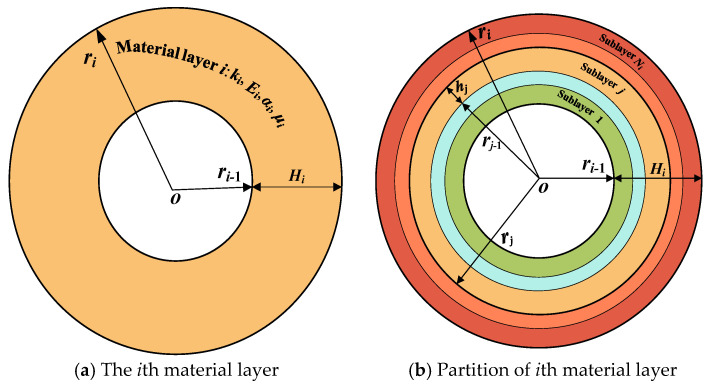
The slice model for the *i*th material layer.

**Figure 4:**
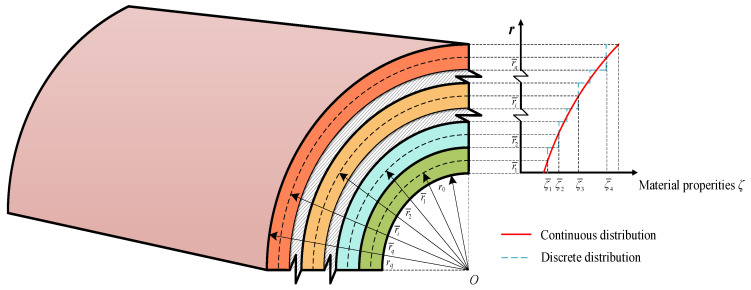
Discretization of the *r*-dependent parameters along the thickness direction.

On account of the temperature-dependent material properties, the temperature distribution along the *r*-direction is nonuniform, which results in the nonuniformity of the material in each layer. A slice model is proposed to study the material properties for each layer in the shell throughout the *r*-direction. As illustrated in Figure 2 and Figure 3, each material layer in the laminated cylindrical shell is classified into a series of sublayers *N_i_* (*i* = 1, 2, …, *q*), totaling *N* layers (*N* = *N*_1_ + *N*_2_ + …+*N_q_*). The thickness of the sublayer is expressed by *h_i_* (*i* = 1, 2, …, *N*). If the sublayer is sufficiently thin, it can be assumed that the material properties of every sublayer remain consistent along the *r*-direction, and the material property at the mid-plane *r* = ${\bar{r}}_{i}$ can be utilized to represent the material properties of the entire sublayer, as shown in Figure 4. The error *e* can be eliminated by analyzing the structural stratification strategy.

#### 3.2.2. The Iterative Method

**Figure 5:**
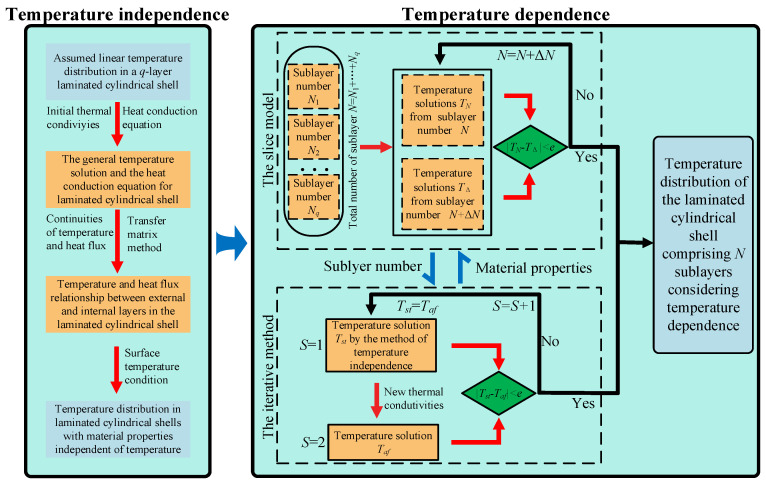
The flowchart of temperature analysis for temperature dependence.

The material properties of each layer are associated with variations in temperature, leading to variations in these properties as the temperature changes. Concurrently, these property modifications also have a reciprocal influence on the temperature distribution throughout the structural domain. An iterative method is introduced to analyze the distribution of temperature within the temperature-dependent shell, as shown in Figure 5. Without the loss of generality, the initial temperature distribution can first be considered to vary linearly along the *r*-direction, obtained from the temperature conditions at internal and external surfaces of the laminated shell. The thermal conductivities of each sublayer are sequentially obtained based on the initial temperature field and the slice model, in accordance with the thermal conductivity–temperature relationship. The new temperature distribution can be accurately obtained based on the updated material properties, as detailed in Section 3.1. This initial iterative step is denoted as *s* = 1. Due to the temperature-dependent structural material, the thermal conductivity in each sublayer varies with the prevailing temperature field and can be recalculated accordingly. Subsequently, updated temperature solutions can be derived, with the iterative step being denoted as *s* = 2. By analogy with this approach, the final temperature distribution can be determined uniquely when the error *e* resulting from the iterative method is eliminated by comparing the consecutive algorithm steps *s* = *j* + 1 with *s* = *j* (*j* = 1, 2, …).

## 4. Solutions for Stresses and Displacements

### 4.1. Basic Equations

The equilibrium differential equation for the axisymmetric laminated cylindrical shell is given in [39]

(11) $$\begin{array}{c} \frac{\partial \sigma_{z}^{i}\left(r,z\right)}{\partial z}+\frac{\partial \tau_{rz}^{i}\left(r,z\right)}{\partial r}+\frac{\tau_{rz}^{i}\left(r,z\right)}{r}=0,\;\frac{\partial \tau_{rz}^{i}\left(r,z\right)}{\partial z}+\frac{\partial \sigma_{r}^{i}\left(r,z\right)}{\partial r}+\frac{\sigma_{r}^{i}\left(r,z\right)-\sigma_{\theta }^{i}\left(r,z\right)}{r}=0, \end{array}$$

where $\sigma_{z}^{i}\left(r,z\right)$ and $\sigma_{r}^{i}\left(r,z\right)$ are the normal stresses and $\tau_{rz}^{i}\left(r,z\right)$ is the shear stress.

The geometric relationships for the cylindrical shells can be stated as

(12) $$\begin{array}{cc} \varepsilon_{r}^{i}\left(r,z\right)=\frac{\partial w_{i}\left(r,z\right)}{\partial r}, & \;\varepsilon_{\theta }^{i}\left(r,z\right)=\frac{w_{i}\left(r,z\right)}{r},\;\\ \varepsilon_{z}^{i}\left(r,z\right)=\frac{\partial u_{i}\left(r,z\right)}{\partial z},\; & \;\gamma_{rz}^{i}\left(r,z\right)=\frac{\partial w_{i}\left(r,z\right)}{\partial z}+\frac{\partial u_{i}\left(r,z\right)}{\partial r}, \end{array}$$

where $\varepsilon_{r}^{i}\left(r,z\right)$, $\varepsilon_{\theta }^{i}\left(r,z\right)$, and $\varepsilon_{z}^{i}\left(r,z\right)$ are the normal strain of the *i*th layer, $\gamma_{rz}^{i}\left(r,z\right)$ is the shear strain, $w_{i}\left(r,z\right)$ is the radial displacement, and $u_{i}\left(r,z\right)$ is the axial displacement.

The thermoelastic constitutive relationships for the cylindrical shell can be expressed as

(13) $$\left\{\begin{array}{l} \sigma_{r}^{i}\left(r,z\right)\\ \sigma_{z}^{i}\left(r,z\right)\\ \sigma_{\theta }^{i}\left(r,z\right)\\ \tau_{rz}^{i}\left(r,z\right) \end{array}\right\}=\left[\begin{array}{cccc} \lambda_{i}+2G_{i} & \lambda_{i} & \lambda_{i} & 0\\ \lambda_{i} & \lambda_{i}+2G_{i} & \lambda_{i} & 0\\ \lambda_{i} & \lambda_{i} & \lambda_{i}+2G_{i} & 0\\ 0 & 0 & 0 & G_{i} \end{array}\right]\left\{\begin{array}{c} \varepsilon_{r}^{i}\left(r,z\right)\\ \varepsilon_{z}^{i}\left(r,z\right)\\ \varepsilon_{\theta }^{i}\left(r,z\right)\\ \gamma_{rz}^{i}\left(r,z\right) \end{array}\right\}-\left\{\begin{array}{c} t_{i}\left(r\right)\\ t_{i}\left(r\right)\\ t_{i}\left(r\right)\\ 0 \end{array}\right\},$$

where *λ_i_* and *G_i_* are Lamè constants for the homogeneous and isotropic material, as follows:

(14) $$\lambda_{i}=\frac{\mu_{i}E_{i}}{\left(1+\mu_{i}\right)\left(1-2\mu_{i}\right)},\;G_{i}=\frac{E_{i}}{2\left(1+\mu_{i}\right)},$$

and $t_{i}\left(r\right)$ can be denoted as

(15) $$t_{i}\left(r\right)=\left(3\lambda_{i}+2G_{i}\right)T_{i}\left(r\right)\alpha_{i}.$$

### 4.2. Solutions for Stresses and Displacements Within the Single-Layer Shell

Based on basic Equations (11)–(13), the state equation can be established by employing $u_{i}\left(r,z\right)$, $\sigma_{r}^{i}\left(r,z\right)$, $\gamma_{rz}^{i}\left(r,z\right)$, and $w_{i}\left(r,z\right)$ as state variables, as follows:

(16) $$\frac{\partial R_{i}\left(r,z\right)}{\partial r}=D\left(r,z\right)R_{i}\left(r,z\right)+B_{i}\left(r,z\right),$$

with

(17) $$\begin{array}{c} R_{i}\left(r,z\right)=\left\{\begin{array}{c} u_{i}\left(r,z\right)\\ \sigma_{r}^{i}\left(r,z\right)\\ \tau_{rz}^{i}\left(r,z\right)\\ w_{i}\left(r,z\right) \end{array}\right\},\;B_{i}\left(r,z\right)=\left\{\begin{array}{c} 0\\ \frac{1-2\mu_{i}}{\left(\mu_{i}-1\right)r}t_{i}\left(r\right)\\ \frac{2\mu_{i}-1}{\mu_{i}-1}\rho t_{i}\left(r\right)\\ \frac{\left(1+\mu_{i}\right)\left(1-2\mu_{i}\right)}{E_{i}\left(1-\mu_{i}\right)}t_{i}\left(r\right) \end{array}\right\},\\ D\left(r,z\right)=\left[\begin{array}{cccc} 0 & 0 & \frac{2\left(1+\mu_{i}\right)}{E_{i}} & -\rho \\ \frac{\mu_{i}E_{i}}{\left(1-{\mu_{i}}^{2}\right)r}\rho & \frac{1-2\mu_{i}}{\left(\mu_{i}-1\right)r} & -\rho & \frac{E_{i}}{\left(1-{\mu_{i}}^{2}\right)r^{2}}\\ \frac{E_{i}}{{\mu_{i}}^{2}-1}\rho^{2} & \frac{\mu_{i}}{\mu_{i}-1}\rho & -\frac{1}{r} & \frac{\mu_{i}E_{i}}{\left({\mu_{i}}^{2}-1\right)r}\rho \\ \frac{\mu_{i}}{\mu_{i}-1}\rho & \frac{\left(1+\mu_{i}\right)\left(1-2\mu_{i}\right)}{E_{i}\left(1-\mu_{i}\right)} & 0 & \frac{\mu_{i}}{\left(\mu_{i}-1\right)r} \end{array}\right], \end{array}$$

where *ρ* = *∂*/*∂z* denotes the partial differential operator concerning *z.*

The boundary conditions at the edges of the simply supported laminated cylindrical shell are

(18) $$w_{i}\left(r,0\right)=w_{i}\left(r,L\right)=0,\;\sigma_{z}^{i}\left(r,0\right)=\sigma_{z}^{i}\left(r,L\right)=0.$$

According to the boundary conditions, the displacements and stresses can be described as

(19) $$\begin{array}{c} u_{i}\left(r,z\right)=\sum_{m=1}^{\infty }U_{i}^{m}\left(r\right)\cos\left(\eta_{m}z\right),\;w_{i}\left(r,z\right)=\sum_{m=1}^{\infty }W_{i}^{m}\left(r\right)\sin\left(\eta_{m}z\right),\\ \sigma_{r}^{i}\left(r,z\right)=\sum_{m=1}^{\infty }Y_{i}^{m}\left(r\right)\sin\left(\eta_{m}z\right),\;\tau_{rz}^{i}\left(r,z\right)=\sum_{m=1}^{\infty }X_{i}^{m}\left(r\right)\cos\left(\eta_{m}z\right), \end{array}$$

where *η_m_* = *mπ*/*L*, $U_{i}^{m}\left(r\right)$, and $W_{i}^{m}\left(r\right)$ are undetermined displacement functions, $Y_{i}^{m}\left(r\right)$ and $X_{i}^{m}\left(r\right)$ are undetermined stress functions.

Based on the slice model, the mean radius $\bar{r_{i}}$ at the mid-plane of each sublayer along the thickness direction can be used to replace the radius *r* in the coefficient matrix of the state Equation (17), provided that the sublayer is sufficiently thin. Substituting the general solutions (19) into the state Equation (16) leads to

(20) $$\frac{dR_{i}^{m}\left(r\right)}{dr}=D_{i}^{m}R_{i}^{m}\left(r\right)+B_{i}^{m}(r),$$

where

(21) $$\begin{array}{c} R_{i}^{m}\left(r\right)=\left\{\begin{array}{c} U_{i}^{m}\left(r\right)\\ Y_{i}^{m}\left(r\right)\\ X_{i}^{m}\left(r\right)\\ W_{i}^{m}\left(r\right) \end{array}\right\},\;B_{i}^{m}\left(r\right)=\left\{\begin{array}{c} 0\\ \frac{1-2\mu_{i}}{\left(\mu_{i}-1\right){\bar{r}}_{i}}T_{i}^{m}\left(r\right)\\ \frac{2\mu_{i}-1}{\mu_{i}-1}\eta_{m}T_{i}^{m}\left(r\right)\\ \frac{\left(1+\mu_{i}\right)\left(1-2\mu_{i}\right)}{E_{i}\left(1-\mu_{i}\right)}T_{i}^{m}\left(r\right) \end{array}\right\},\\ D_{i}^{m}=\left[\begin{array}{cccc} 0 & 0 & \frac{2\left(1+\mu_{i}\right)}{E_{i}} & -\eta_{m}\\ \frac{\mu_{i}E_{i}}{\left({\mu_{i}}^{2}-1\right){\bar{r}}_{i}}\eta_{m} & \frac{1-2\mu_{i}}{\left(\mu_{i}-1\right){\bar{r}}_{i}} & \eta_{m} & \frac{E_{i}}{\left(1-{\mu_{i}}^{2}\right){{\bar{r}}_{i}}^{2}}\\ \frac{E_{i}}{1-{\mu_{i}}^{2}}\eta_{m}^{2} & \frac{\mu_{i}}{\mu_{i}-1}\eta_{m} & -\frac{1}{{\bar{r}}_{i}} & \frac{\mu_{i}E_{i}}{\left({\mu_{i}}^{2}-1\right){\bar{r}}_{i}}\eta_{m}\\ \frac{\mu_{i}}{1-\mu_{i}}\eta_{m} & \frac{\left(1+\mu_{i}\right)(1-2\mu_{i})}{E_{i}\left(1-\mu_{i}\right)} & 0 & \frac{\mu_{i}}{\left(\mu_{i}-1\right){\bar{r}}_{i}} \end{array}\right], \end{array}$$

where

(22) $$T_{i}^{m}\left(r\right)=\frac{2}{L}\int_{0}^{L}t_{i}\left(r\right)\sin\left(\eta_{m}z\right)dz.$$

The solution of the state Equation (20) for each sublayer can be formulated as follows:

(23) $$R_{i}^{m}\left(r\right)=G_{i}^{m}\left(r-r_{i-1}\right)R_{i}^{m}\left(r_{i-1}\right)+C_{i}^{m}\left(r-r_{i-1}\right),$$

where

(24) $$G_{i}^{m}\left(r-r_{i-1}\right)=\exp\left[D_{i}^{m}\cdot \left(r-r_{i-1}\right)\right],\;C_{i}^{m}\left(r-r_{i-1}\right)=\int_{r_{i-1}}^{r}\exp\left[D_{i}^{m}\cdot \left(r-r_{i-1}\right)\right]B_{i}^{m}\left(\tau \right)d\tau .$$

The induced variable $\sigma_{z}^{i}\left(r,z\right)$ can be ascertained based on the state variables, as follows:

(25) $$\sigma_{z}^{i}\left(r,z\right)=\frac{E_{i}}{1-\mu_{i}^{2}}\frac{\partial }{\partial z}u_{i}\left(r,z\right)-\frac{\mu_{i}}{\mu_{i}-1}\sigma_{r}^{i}\left(r,z\right)+\frac{\mu_{i}E_{i}}{(1-\mu_{i}^{2})r}w_{i}\left(r,z\right)-\frac{2\mu_{i}-1}{\mu_{i}-1}t_{i}\left(r\right).$$

### 4.3. Recursive Formulas for Stresses and Displacements

Taking *r* = *r_i_* into the mechanical solution (23), the relationships of the stresses and displacements between the outer and inner surfaces of the *i*th layer are obtained as follows:

(26) $$R_{i}^{m}\left(r_{i}\right)=G_{i}^{m}\left(r_{i}-r_{i-1}\right)R_{i}^{m}\left(r_{i-1}\right)+C_{i}^{m}\left(r_{i}-r_{i-1}\right)\;\;\;(i=1,2,\ldots ,N).$$

The displacements and stresses are continuous at the interfaces, i.e.,

(27) $$R_{i+1}^{m}\left(r_{i}\right)=R_{i}^{m}\left(r_{i}\right),\;\;\;(i=1,2,\ldots ,N-1).$$

On the basis of Equations (26) and (27), the relationships for stresses and displacements between the first sublayer and the *p*th (*p* = 2, 3, …, *N*) sublayer can be derived recursively as

(28) $$R_{p}^{m}(r_{p})=\left[\prod_{i=p}^{1}G_{i}^{m}\left(h_{i}\right)\right]R_{1}^{m}\left(r_{0}\right)+\sum_{j=1}^{p-1}\left\{\left[\prod_{i=p}^{j+1}G_{i}^{m}\left(h_{i}\right)\right]C_{j}^{m}\left(h_{j}\right)\right\}+C_{p}^{m}\left(h_{p}\right),$$

where *h_i_* = *r_i_* − *r_i_*_−1_, (*i* = 1, 2, …, *N*).

Substituting the sum of the sublayer *N* into Equation (28), the relationships for displacements and stresses between the external layer and internal layer of the laminated shell can be determined as

(29) $$R_{N}^{m}\left(r_{N}\right)=\Lambda R_{1}^{m}\left(r_{0}\right)+\bar{\Lambda },$$

where

(30) $$\Lambda =\prod_{i=N}^{1}G_{i}^{m}\left(h_{i}\right),\;\;\bar{\Lambda }=\sum_{j=1}^{N-1}\left\{\left[\prod_{i=N}^{j+1}G_{i}^{m}\left(h_{i}\right)\right]C_{j}^{m}\left(h_{j}\right)\right\}+C_{N}^{m}\left(h_{N}\right).$$

### 4.4. Exact Solutions for Stresses and Displacements

The mechanical loads acting on the surface are $Q_{in}\left(z\right)$ and $Q_{ex}\left(z\right)$, respectively. The stress conditions on the surface can be expressed as

(31) $$\tau_{rz}^{1}\left(r_{0},z\right)=\tau_{rz}^{N}\left(r_{N},z\right)=0,\;\;\sigma_{r}^{1}\left(r_{0},z\right)=Q_{in}\left(z\right),\;\;\sigma_{r}^{N}\left(r_{N},z\right)=Q_{ex}\left(z\right).$$

Substituting the stress conditions (31) into the general solution (19) yields

(32) $$\begin{array}{c} X_{N}^{m}\left(r_{N}\right)=X_{1}^{m}\left(r_{0}\right)=0,\\ Y_{1}^{m}\left(r_{0}\right)=-\frac{2Q_{in}\left(z\right)}{m\pi }\left[1-{\left(-1\right)}^{m}\right],\\ Y_{N}^{m}\left(r_{N}\right)=-\frac{2Q_{ex}\left(z\right)}{m\pi }\left[1-{\left(-1\right)}^{m}\right]. \end{array}$$

Based on the recursive Equation (29), it follows that the displacement functions $U_{1}^{m}\left(r_{0}\right)$ and $W_{1}^{m}\left(r_{0}\right)$ for the internal surface of the shell are given by

(33) $$\left[\begin{array}{c} U_{1}^{m}\left(r_{0}\right)\\ W_{1}^{m}\left(r_{0}\right) \end{array}\right]={\left[\begin{array}{cc} \Lambda_{21} & \Lambda_{24}\\ \Lambda_{31} & \Lambda_{34} \end{array}\right]}^{-1}\left\{\left.\left[\begin{array}{c} Y_{N}^{m}\left(r_{N}\right)\\ X_{N}^{m}\left(r_{N}\right) \end{array}\right]-\left[\begin{array}{cc} \Lambda_{22} & \Lambda_{23}\\ \Lambda_{32} & \Lambda_{33} \end{array}\right]\left[\begin{array}{c} Y_{1}^{m}\left(r_{0}\right)\\ X_{1}^{m}\left(r_{0}\right) \end{array}\right]-\left[\begin{array}{c} {\bar{\Lambda }}_{2}\\ {\bar{\Lambda }}_{3} \end{array}\right]\right\}\right..$$

By substituting the stress boundary conditions (32) into Equation (33), the unknown displacement functions of the internal surface $U_{1}^{m}\left(r_{0}\right)$ and $W_{1}^{m}\left(r_{0}\right)$ can be determined. Taking the displacements $U_{1}^{m}\left(r_{0}\right)$ and $W_{1}^{m}\left(r_{0}\right)$, and the stresses $Y_{1}^{m}\left(r_{0}\right)$ and $X_{1}^{m}\left(r_{0}\right)$ of the internal surface into the recursive relationship (28), the state variable $R_{p}^{m}(r_{p})$ of the *p*th layer (*p* = 1, 2, …, *N*) can be obtained. Based on continuities at the interfaces (27), the value of displacements and stresses at any location in the laminated shell with temperature-dependent materials can be finalized uniquely by taking the $R_{p}^{m}(r_{p})$ into the solution of the state Equation (23) and the general solution (19).

## 5. Convergence and Comparison Studies

This section validates the accuracy and convergence of this approach through an analysis of displacements and stresses in a three-layer laminated shell under temperature and mechanical loads. The surface of the cylindrical shell is steel, and the core layer is concrete. The material properties discussed in this paper are outlined in Table 1 and Table 2 [40,41]. The radii of each material layer are as follows: *r*_0_ = 1.0 m, *r*_1_ = 1.3 m, *r*_2_ = 1.9 m, and *r*_3_ = 2.2 m. The length is *L* = 6 m. The internal surface temperature is *t*_0_ = 20 °C and the external surface temperature is *t_q_* = 100 °C. The mechanical load acting on the internal surface is *Q_in_*(*z*) = 20 kN/m^2^.

The number of the sublayers *N* and the iterative steps *s* in the iterative approach are discussed to accurately determine the temperature solutions for laminated shells with temperature-dependent materials. Table 3 presents the temperature solutions at five arbitrary points along *z* = 2.0 m, *r* = 1.15 m, 1.45 m, 1.60 m, 1.75 m, and 2.05 m, respectively. The data from different numbers of the sublayers *N* = 12, 16, 20, and 24 and iterative steps *s* = 1, 2, 3, 4, and 5 have been thoroughly examined. As evidenced in Table 3, the computing results are accurate to four decimal places when the number of sublayers *N* ≥ 20 and the iterative steps *s* ≥ 3.

The sublayer number *N* and the truncated terms *M* have been carefully examined to capture accurate solutions for displacements and stresses. The results at an arbitrary point *r* = 1.15 m, *z* = 1.4 m under sublayer numbers *N* = 12, 16, 20, and 24 and truncated terms *M* = 10, 20, 30, 40, and 50 are shown in Table 4. The data demonstrate rapid convergence as the truncated terms *M* and the sublayer numbers *N* increase. The results for displacement and stress with *M* = 40 and *N* = 20 are the same as those with *M* = 50 and *N* = 24 up to three significant digits. Therefore, the truncated terms *M* = 40, the number of sublayers *N* = 20, and iterative steps *s* = 3 can be utilized for the subsequent calculations.

To validate the accuracy of the proposed method, a comprehensive finite element analysis (FE) was conducted using ANSYS 2021R1 software. The geometry of the laminated cylindrical shell was modeled according to the given dimensions: inner radius *r*_0_ = 1.0 m, *r*_1_ = 1.3 m, *r*_2_ = 1.9 m, and *r*_3_ = 2.2 m. The length of the shell was set to *L* = 6 m. The internal surface temperature was defined as *t*_0_ = 20 °C, while the external surface temperature was set at *t_q_* = 100 °C. A mechanical load *Q_in_*(*z*) = 20 kN/m^2^ was applied to the internal surface. The boundary conditions assumed simply supported constraints at both ends of the shell. Mesh generation utilized the Volume Sweep (VSweep) technique available in ANSYS to produce high-quality hexahedral elements, particularly suitable for complex geometries with layered structures. The solid226 element type was chosen due to its capability to accurately capture thermal and structural behaviors under coupled loading conditions. An initial global element size of 0.1 m × 0.1 m was specified for the entire domain, with local refinement applied near areas of high gradient, such as interfaces between material layers. Mesh quality was assessed by evaluating parameters like aspect ratio, skewness, and orthogonality, ensuring all elements met predefined quality standards for reliable numerical results. A mesh convergence study was performed to confirm that the solution is independent of mesh density. Mesh iterations were refined until the relative change in key variables (temperature, displacement, and stress) was less than 1%. Material properties for each layer were defined according to data provided in Table 1, incorporating temperature-dependent characteristics into the model to account for realistic behavior under varying thermal conditions. Thermal boundary conditions included a fixed temperature of *t*_0_ = 20 °C on the internal surface and *t_q_* = 100 °C on the external surface. Mechanical boundary conditions involved applying a pressure load of 20 kN/m^2^ on the internal surface, with simply supported boundary conditions applied to both ends of the shell. The FE was conducted in two stages: thermal analysis, utilizing Fourier’s law of heat conduction to calculate the temperature distribution within the shell, and structural analysis, coupling the obtained temperature field with the structural solver to compute displacements and stresses. The finite element solutions were compared against the analytical results obtained from the proposed method. Table 5 summarizes the temperature, displacement, and stress solutions at five arbitrary points within the shell (*r* = 1.15, 1.45 m, 1.60 m, 1.75 m, and 2.05 m at *z* = 1.60 m). As shown in Table 5, the FE solutions closely matched the analytical outcomes, validating the accuracy of the proposed approach. It can be observed that the FE solutions perfectly match the solutions of the present method.

**Figure 6:**
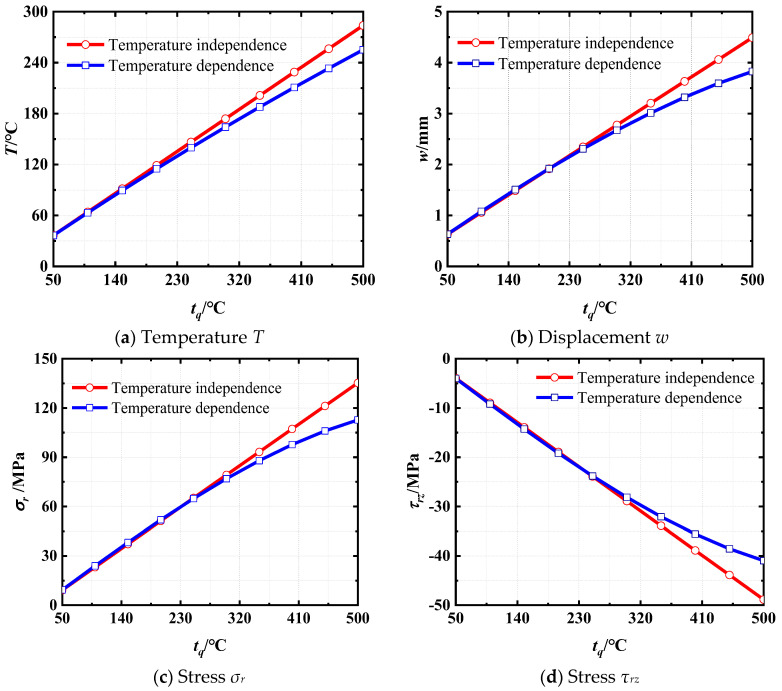
Comparisons of *T*, *w*, *σ_r_*, and *τ_rz_* at *r* = 1.6 m, *z* = 2.0 m between temperature dependence and temperature independence under different surface temperatures.

Furthermore, the impact of the coupled interaction between material properties and temperature variations is investigated on both the displacement and stress distributions within the structure under a thermal environment. Figure 6 illustrates the comparative analysis of temperature, displacements, and stresses under disparate surface temperatures at an arbitrary point (*r* = 1.6 m, *z* = 2.0 m) for both cases: temperature dependence and temperature independence. It can be observed that the difference between the two states becomes increasingly larger as the environment’s temperature increases. Ultimately the difference in temperature escalates to 11.28%, the difference in displacement expands to 17.35%, and the difference in stress significantly increases to 33.74%. Therefore, it is essential to consider the temperature-dependent material properties for an accurate analysis of the mechanical behavior of laminated cylindrical shells under the combined temperature and mechanical loads.

## 6. Parametric Analysis

This section presents three numerical analyses to evaluate the impact of surface temperature, thickness-to-radius ratios, layer numbers, and component materials on temperature, stresses, and displacements in cylindrical shells under thermo-mechanical loads. The analysis accounts for the interactions of temperature and material properties.

### 6.1. Influence of Surface Temperature

Temperature, displacement, and stress distributions within a three-layer shell under different temperature environments are investigated. The internal and external layers of the shell are made of steel. The core layer is made of concrete. The length is *L* = 10 m. The radii of material layers are *r*_0_ = 1.00 m, *r*_1_ = 1.05 m, *r*_2_ = 1.15 m, and *r*_3_ = 1.20 m, respectively. The internal surface temperature is *t*_0_ = 20 °C, while the external surface temperature is designated as *t_q_* = 100 °C, 300 °C, and 500 °C, respectively. Furthermore, a distributed load *Q_in_*(*z*) = 10 kN/m^2^ acts on the internal surface of the shell.

**Figure 7:**
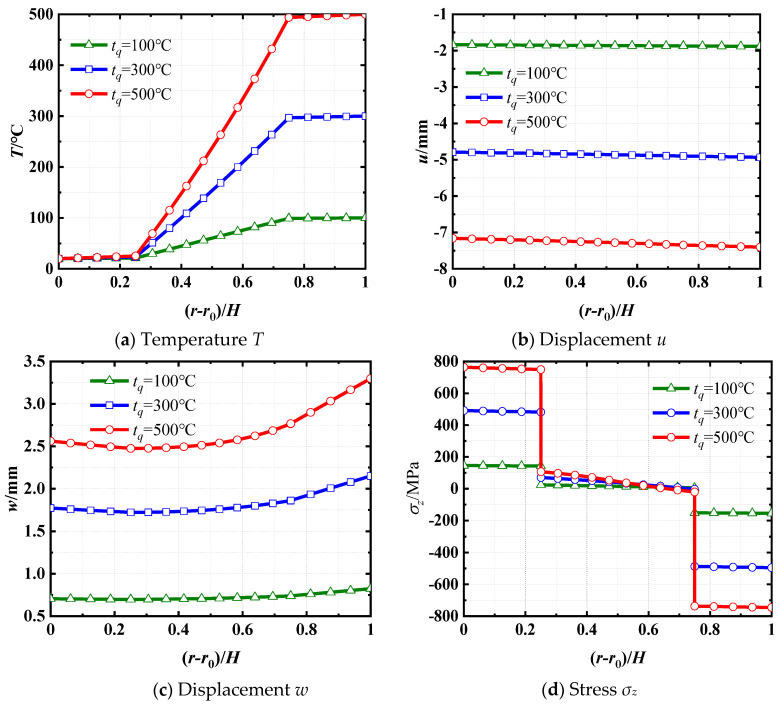
Distributions of *T*, *u*, *w*, and *σ_z_* at *z* = 2.5 m along the thickness direction for three different surface temperatures.

The distributions of the temperature *T*, axial displacement *u*, radial displacement *w*, and axial stress *σ_z_* along the *r*-direction at *z* = 2.5 m are presented in Figure 7. Figure 7a demonstrates that the temperature *T* in each layer increases with the surface temperature, but not linearly. This is due to the thermal conductivity of each layer varying with temperature. As illustrated in Figure 7b, the absolute value of axial displacement *u* remains relatively constant along the *r*-direction. Furthermore, an increase in surface temperature results in an increase in displacement *u* at the same point. Figure 7c illustrates that the radial displacement *w* increases with the surface temperature, and the variation in displacement along the *r*-direction becomes more pronounced as the thermal load increases. The effect is a result of the thermal expansion coefficient increasing as surface temperature rises. In Figure 7d, it can be observed that the axial stress *σ_z_* in each layer increases with increasing surface temperature. Additionally, the stress *σ_z_* at the boundaries between adjacent layers exhibits abrupt changes, and this difference increases with the surface temperature.

**Figure 8:**
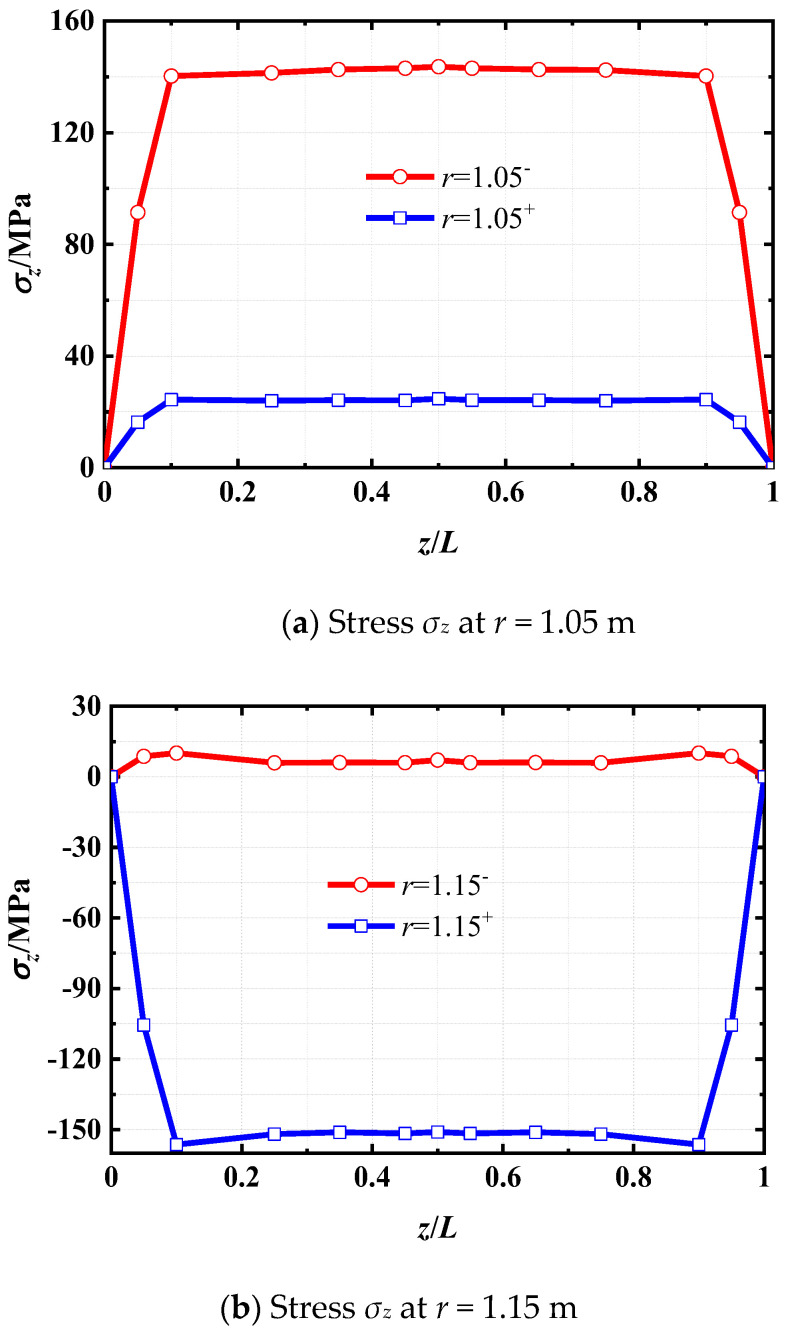
Distribution of *σ_z_* at *r* = 1.05 m and *r* = 1.15 m along the *z*-direction for *t_q_* = 100 °C. Note: the superscript “−” means the inner surface of the interface and the superscript “+” means the outer surface of the interface.

To delve deeper into the investigation of interface stress *σ_z_*, the stress distributions along the *z*-direction at both interfaces *r* = 1.05 m and *r* = 1.15 m in the laminated shell, under the surface temperature *t_q_* = 100 °C, are presented in Figure 8. It can be noticed that the axial stress *σ_z_* in the steel layer consistently exceeds that in the concrete layer. This disparity is attributed to the significantly higher elastic modulus of steel compared to concrete.

### 6.2. Influence of Thickness-to-Radius Ratios H/r_0_

Three simply supported three-layer shells with different internal radii *r*_0_ = 0.25 m, 0.50 m, and 1.00 m are investigated to analyze the effect of thickness-to-radius ratios on the distributions of temperature, stress, and displacement. The thickness of all three shells is *H* = 0.2 m (*H*_1_ = *H*_3_ = 0.05 m, *H*_2_ = 0.1 m), i.e., *H*/*r*_0_ = 0.8, 0.4, and 0.2, respectively. The length is *L* = 8 m. The material of the surface layers is steel, and the core layer is made of concrete. The internal temperature is *t*_0_ = 20 °C, while the external temperature is *t_q_* = 200 °C for the laminated cylindrical shell. The external shell surface is stressed with a pressure load *Q_ex_* (*z*) = 15 kN/m^2^.

**Figure 9:**
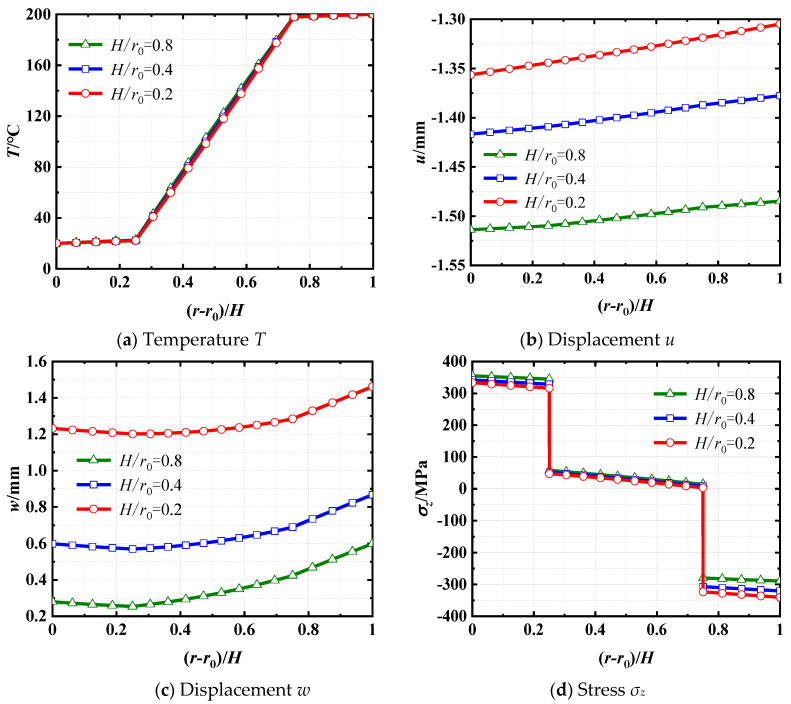
Distributions of *T*, *u*, *w*, and *σ_z_* at *z* = 3 m along the thickness direction for three different thickness-to-radius ratios.

The distributions of temperature *T*, axial displacement *u*, radial displacement *w*, and axial stress *σ_z_* along the *r*-direction at *z* = 3 m are shown in Figure 9. Figure 9a illustrates that the distribution of temperature in the cylindrical shell is marginally affected by the thickness-to-radius ratios. The temperature distributions are almost identical along the *r*-direction in every layer, regardless of the disparate thickness-to-radius ratios. From Figure 9b,c, it can be noticed that the axial displacement *u* and radial displacement *w* exhibit a decrease as the increasing thickness-to-radius ratio. As illustrated in Figure 9d, the axial stress *σ_z_* variation in the layer made of steel is greater than the concrete layer under different thickness–radius ratios. This is attributed to the elastic modulus of steel being much higher than that of concrete.

### 6.3. Influence of Layer Numbers and Component Materials

Three laminated shells are investigated to study the thermo-mechanical behaviors caused by different layer numbers and materials. The three shells exhibit identical geometrical dimensions. The length is *L* = 6 m. The internal radius *r*_0_ = 1.00 m. The thickness *H* = 0.08 m. The first shell is a two-layer shell with the radii *r*_1_ = 1.04 m and *r*_2_ = 1.08 m. The structure consists of an internal concrete layer and an external steel layer. The second shell is a three-layer shell with the radii *r*_1_ = 1.02 m, *r*_2_ = 1.06 m, and *r*_3_ = 1.08 m. The surface layer of the shell is concrete and the core layer is steel. The third shell is a four-layer shell with the radii *r*_1_ = 1.02 m, *r*_2_ = 1.04 m, *r*_3_ = 1.06 m and *r*_4_ = 1.08 m. The material properties of the internal and third layers are steel, while the external and second layers are made of concrete. The temperatures on the surfaces of all three shells are *t*_0_ = 20 °C and *t_q_* = 400 °C, respectively. The internal surfaces of the shells are subjected to an identical internal pressure *Q_in_*(*z*) = 20 kN/m^2^.

**Figure 10:**
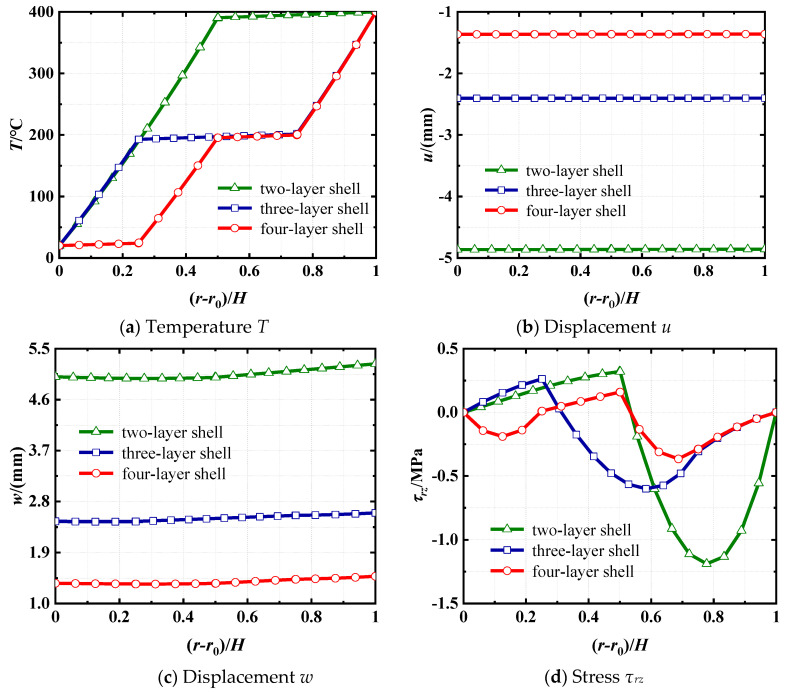
Distributions of *T*, *u*, *w*, and *τ_rz_* at *z* = 2 m along the thickness direction for different layer numbers and component materials.

The distributions of temperature *T*, axial displacement *u*, radial displacement *w*, and shear stress *τ_rz_* at *z* = 2 m along the *r*-direction are shown in Figure 10. It is evident that, given the same geometric dimensions, temperature loads, and internal pressure for the shell, the material properties and the number of layers significantly affect the temperatures, displacements, and stresses. Figure 10a shows that the temperature variations within the concrete layer are always greater than that of the steel layer. This is because steel exhibits significantly greater thermal conductivity compared to concrete. As illustrated in Figure 10b,c, the axial displacement *u* and radial displacement *w* vary very little along the *r*-direction. Additionally, the value of displacements at the same location decreases significantly in absolute value with an increasing number of layers. Figure 10d shows that the maximum values of absolute shear stress *τ_rz_* always appear in the steel layer for all three shells. This is because the elastic modulus for concrete is considerably less than that of steel. Additionally, the maximum value decreases as the number of layers increases. Thus, the temperature, stress, and displacement distributions in laminated shells under thermo-mechanical loads can be designed and optimized by adjusting the number of layers and the materials used. This insight offers a valuable reference for ensuring the structural safety and design of laminated cylindrical shells operating under temperature loads.

## 7. Conclusions

This paper investigated the mechanical behaviors of laminated cylindrical shells subjected to temperature and mechanical loads. By applying Fourier’s law of heat conduction and the theory of thermoelasticity, exact solutions for temperature, stresses, and displacements were determined. Due to the intricate interaction between variable temperature and material properties, the slice model and the iterative method were introduced to address the temperature dependence of the component materials in the laminated shell. Comparative and convergence analysis validated the accuracy and effectiveness of the proposed approach. Additionally, the influence of temperature dependence on the temperature, displacements, and stresses within the shell was studied through comparative analyses. Furthermore, the effects of the surface temperature, thickness-to-radius ratio, number of layers, and component materials on temperature, stress, and displacement distributions within the shell were explored in depth through three parametric analyses. The results indicated that the absolute values of temperature, stresses, and displacements exhibited a significant increase in correlation with the rise in surface temperature. The axial displacement *u* and radial displacement *w* decreased as the thickness-to-radius ratio increased. The absolute values of stresses and displacements in the two-layer shells were always greater than those in the three-layer and four-layer shells, even when the geometric dimensions and thermo-mechanical loads were the same. These findings highlight the importance of considering both structural configuration and material selection in optimizing performance. Moreover, our research not only advances the systematic modeling and analysis of such systems but also provides a robust foundation for future studies in the field. Practical applications include design optimization, enhanced understanding of material behavior under thermal–mechanical loads, and improved thermal load management strategies. This is particularly significant in high-stress environments like aerospace and energy systems, where reliability and performance are paramount. Consequently, the proposed approach can be effectively applied to optimize the design of laminated cylindrical shells under combined temperature and mechanical loads, ensuring both performance and reliability.

## Figures and Tables

**Table 1:** Material properties of steel and concrete.

Materials	*k_i_* [W/(m°C)]	*μ_i_*	*α_i_* (°C^−1^)
Steel	−3.33 × 10^−2^ *T* + 54	0.3	4 × 10^−9^ *T*^2^ + 1.208 × 10^−5^
Concrete	5.7 × 10^−7^ *T*^2^ − 1.36 × 10^−3^ *T* + 1.36	0.2	1.4 × 10^−11^ *T*^2^ + 2.8 × 10^−10^ *T* + 6.0056 × 10^−6^

**Table 2:** The elastic moduli of steel and concrete.

*T* (°C)	Steel (GPa)	Concrete (GPa)
20	210	30
100	210	30
200	189	-
300	169	-
400	147	-
500	126	-
600	65.1	-
700	27.3	0

Note: Linear interpolation was used to evaluate the elastic moduli.

**Table 3:** Temperature solutions (°C) at *z* = 2.0 m for different iterative steps *s* and sublayer number *N*.

Position	*N*_1_	*N*_2_	*N*_3_	*N*	Iterative Step *s*				
					*s* = 1	*s* = 2	*s* = 3	*s* = 4	*s* = 5
*r* = 1.15 m	3	6	3	12	20.7171	20.6889	20.6892	20.6892	20.6892
	4	8	4	16	20.7171	20.6889	20.6893	20.6893	20.6893
	5	10	5	20	20.7171	20.6889	20.6893	20.6893	20.6893
	6	12	6	24	20.7171	20.6889	20.6893	20.6893	20.6893
*r* = 1.45 m	3	6	3	12	43.7626	43.1245	43.1275	43.1276	43.1276
	4	8	4	16	43.7626	43.0994	43.1029	43.1030	43.1030
	5	10	5	20	43.7626	43.1083	43.1116	43.1117	43.1117
	6	12	6	24	43.7626	43.0993	43.1115	43.1117	43.1117
*r* = 1.60 m	3	6	3	12	63.9704	63.1830	63.1818	63.1819	63.1818
	4	8	4	16	63.9704	63.1827	63.1815	63.1816	63.1816
	5	10	5	20	63.9704	63.1826	63.1814	63.1815	63.1815
	6	12	6	24	63.9704	63.1825	63.1813	63.1815	63.1815
*r* = 1.75 m	3	6	3	12	82.3660	81.8424	81.8384	81.8385	81.8385
	4	8	4	16	82.3660	81.8244	81.8200	81.8200	81.8200
	5	10	5	20	82.3660	81.8243	81.8199	81.8199	81.8199
	6	12	6	24	82.3660	81.8242	81.8198	81.8199	81.8199
*r* = 2.05 m	3	6	3	12	99.6377	99.6338	99.6336	99.6336	99.6336
	4	8	4	16	99.6377	99.6337	99.6335	99.6335	99.6335
	5	10	5	20	99.6377	99.6337	99.6335	99.6335	99.6335
	6	12	6	24	99.6377	99.6337	99.6335	99.6335	99.6335

**Table 4:** Results for *u*, *σ_r_*, *w*, and *τ_rz_* at the point (*r* = 1.15 m, *z* = 1.40 m) for different sublayer numbers *N* and truncation coefficients *M*.

Displacements or Stresses	Sublayer Number *N*	*M* = 10	*M* = 20	*M* = 30	*M* = 40	*M* = 50
*u* (mm)	12	−0.814	−0.811	−0.810	−0.810	−0.810
	16	−0.814	−0.811	−0.810	−0.810	−0.810
	20	−0.814	−0.811	−0.810	−0.810	−0.810
	24	−0.814	−0.811	−0.810	−0.810	−0.810
*σ_r_* (MPa)	12	16.4	15.4	14.7	15.3	15.3
	16	16.7	15.7	15.0	15.6	15.6
	20	16.7	15.7	15.0	15.5	15.5
	24	16.7	15.7	15.0	15.5	15.5
*W* (mm)	12	0.815	0.815	0.815	0.815	0.815
	16	0.815	0.815	0.815	0.815	0.815
	20	0.815	0.815	0.815	0.815	0.815
	24	0.815	0.815	0.815	0.815	0.815
*τ_rz_* (MPa)	12	−10.2	−10.2	−10.1	−10.0	−10.0
	16	−10.2	−10.2	−10.1	−10.0	−10.0
	20	−10.2	−10.2	−10.1	−10.0	−10.0
	24	−10.2	−10.2	−10.1	−10.0	−10.0

**Table 5:** Comparisons of temperature, displacements, and stresses between the present solutions and the FE solutions at *z* = 1.60 m.

Position	Method	*T* (°C)	*u* (mm)	*w* (mm)	*σ_z_* (MPa)	*τ_rz_* (MPa)
*r* = 1.15 m	FE	20.7	−0.733	0.823	76.0	−9.77
	present	20.7	−0.733	0.823	75.8	−9.72
*r* = 1.45 m	FE	43.1	−0.920	0.955	19.5	−13.5
	present	43.1	−0.920	0.955	19.5	−13.3
*r* = 1.60 m	FE	63.2	−1.08	1.09	18.8	−12.8
	present	63.2	−1.08	1.09	18.8	−12.7
*r* = 1.75 m	FE	81.8	−1.22	1.24	18.4	−12.1
	present	81.8	−1.22	1.24	18.3	−12.1
*r* = 2.05 m	FE	99.6	−1.38	1.64	−72.5	−7.61
	present	99.6	−1.38	1.64	−72.7	−7.59

## Data Availability

The original contributions presented in the study are included in the article, further inquiries can be directed to the corresponding author.
